# Dysphagia Alone as a Unique Presentation of Wound Botulism in the Emergency Department: A Case Report

**DOI:** 10.5811/cpcem.2020.9.48664

**Published:** 2020-10-20

**Authors:** Ryan H. Wyatt, Eytan Shtull-Leber, Thomas E. Kelly

**Affiliations:** Valleywise Health Medical Center, Department of Emergency Medicine, Phoenix, Arizona

**Keywords:** Wound, botulism, dysphagia

## Abstract

**Introduction:**

Wound botulism is a rare and potentially fatal infectious disease, often seen in patients who abuse injection drugs. It classically presents with dysfunction of bilateral cranial nerves followed by proximal and distal motor weakness, which can progress to respiratory failure.

**Case Report:**

We report a case of a 31-year-old female who presented to the emergency department for the fifth time with an eight-day history of isolated dysphagia without any other neurologic symptoms. She reported a history of injection drug abuse via “skin popping,” was admitted to the hospital, and ultimately diagnosed with wound botulism.

**Conclusion:**

This case exemplifies the diagnostic pitfalls of rare diseases such as wound botulism and provides insight regarding the diagnosis and treatment of this entity. This case also highlights the unique medical and social challenges emergency physicians face while trying to reliably evaluate patients who abuse controlled substances.

## INTRODUCTION

According to the Centers for Disease Control and Prevention (CDC), in 2017 there were 182 laboratory-confirmed cases of botulism reported in the United States, 19 (10%) of which were secondary to wound botulism. Of those 19 patients, none died.^1^ Wound botulism is usually seen in persons who use injection drugs, most commonly using a technique known as “skin popping” (under the skin vs in a vein), or “muscling.”^1^ While wound botulism is a rare entity overall, it does carry a significant morbidity and mortality risk, even if treated.^2^ Wound botulism from injecting drugs is a well-defined entity in the medical literature.^3^,^4^ It is especially common among patients who abuse black tar heroin.^4^

Usually, patients infected with a toxin-producing strain of *Clostridium botulinum* experience cranial nerve dysfunction, commonly affecting more than five cranial nerves.^5^ Symptoms then progress to proximal muscle weakness followed by distal muscle weakness, with respiratory impairment that may require mechanical ventilation. If left untreated, respiratory muscle weakness can lead to respiratory failure and death. We present a case of a patient with isolated dysphagia as a primary symptom, with an initially non-progressive course that later progressed to cranial nerve dysfunction.

## CASE REPORT

A 31-year-old female presented to the emergency department (ED) with an eight-day history of dysphagia, initially to solids then progressing to liquids. The patient denied any other neurologic or gastrointestinal symptoms. The patient had been seen in the ED multiple times for the same complaint, including two visits to other EDs and two prior visits to our ED. During the encounters at the other EDs, she had reportedly had a negative magnetic resonance imaging (MRI) of the brain. On the patient’s initial evaluation in our ED, she denied progressive symptoms and had a neurologic exam that did not reveal any bulbar weakness, ptosis, or gross motor weakness. The patient was noted to have chronic-appearing open wounds to her bilateral upper extremities consistent with a self-reported history of skin-popping/muscling; however, none of the wounds appeared to be acutely infected. After a discussion with radiology, it was felt a swallow study would not be clinically beneficial given her mild symptoms. As she was able to tolerate oral liquids without difficulty, she was discharged home and two days later followed up with neurology.

**Image 1:**
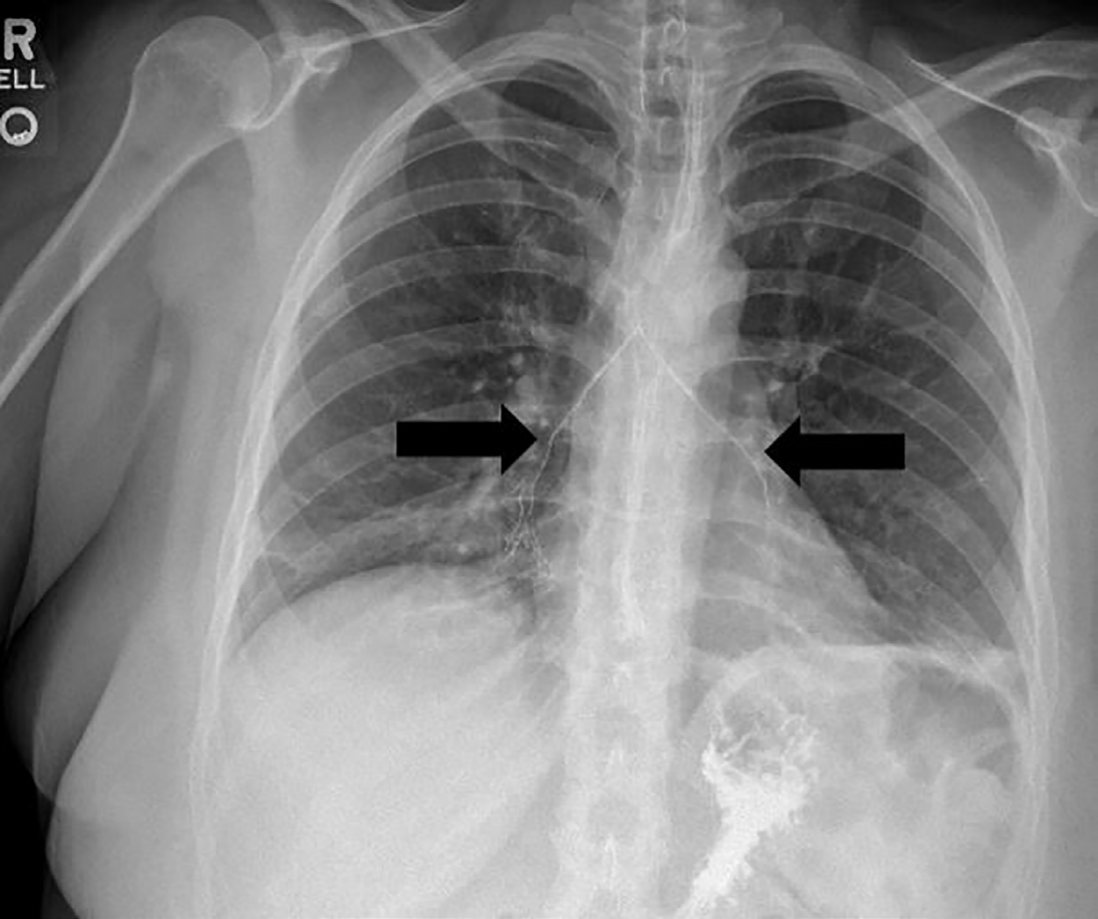
Aspirated contrast material during swallow study of patient diagnosed with wound botulism.

At the neurology follow-up appointment, the patient felt as though her symptoms were progressing. On physical exam the neurologist noted bilateral ptosis and was concerned for progressive bulbar weakness given the evolution of symptoms. She was sent back to the ED for further evaluation and possible MRI but subsequently left without being seen. She returned to the ED later that night and was evaluated again. During that encounter, the patient was found to have ptosis, which could be overcome with effort, while the remainder of her neurologic exam was determined to be normal and non-focal. Laboratory studies in the ED demonstrated a leukocytosis of 13.6 × 10^3^/ microliters (uL) (reference [ref] range 4.0 – 11.0 × 10^3^/uL), mild metabolic acidosis (bicarbonate of 16.0 millimoles (mmol)/ liter (L) (ref range 23 – 30mmol/L), and elevated C-reactive protein of 116.5 milligrams (mg)/L (ref range < 10mg/L). The remainder of her initial labs were normal. Contrast swallow study was obtained, which demonstrated aspiration of contrast material into the trachea that could be visualized in both mainstem bronchi (Image 1).

The patient was admitted to the hospital and the following day was transferred to the intensive care unit (ICU) for progressive respiratory weakness. She was subsequently intubated for airway protection as she had a negative inspiratory force (NIF) of less than −20 centimeters of water. While in the ICU, the patient underwent further study including lumbar puncture and additional imaging. She had normal cerebrospinal fluid examination, negative blood cultures, and normal computed tomography of the head, neck, and chest. She also had normal MRIs of the brain and cervical, thoracic, and lumbar spine. On the second day of hospitalization, differential diagnosis was broadened, and serum, stool, and wound culture samples were sent to the CDC for botulinum testing. The wound tissue sample ultimately tested positive for a toxin producing strain of *C. botulinum*.

CPC-EM Capsule

What do we already know about this clinical entity?

*Wound botulism is a rare, potentially fatal disease process that can present a diagnostic dilemma in the emergency department*.

What makes this presentation of disease reportable?

*This presentation was unique in that the patient presented with isolated dysphagia without other cranial nerve dysfunction*.

What is the major learning point?

*Wound botulism can have a varied presentation, is easy to miss, and should be considered in cases of isolated dysphagia especially among patients who inject heroin*.

How might this improve emergency medicine practice?

*This rare presentation will alert emergency practitioners to expand their own differentials and consider this potentially fatal entity*.

**Image 2:**
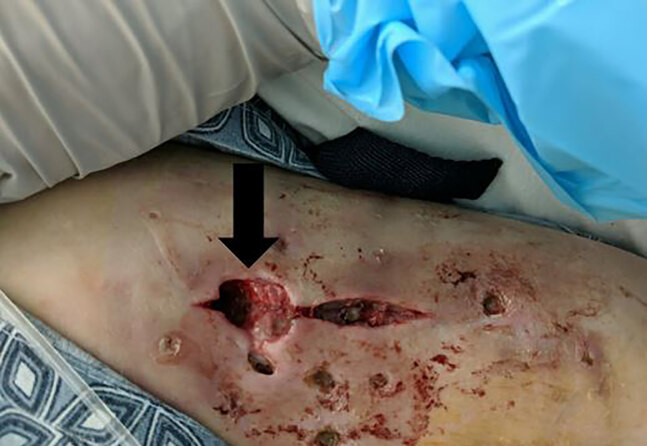
Deep left upper extremity wound from “skin popping.” Photo taken after debridement for wound botulism. Mild surrounding erythema noted.

**Image 3:**
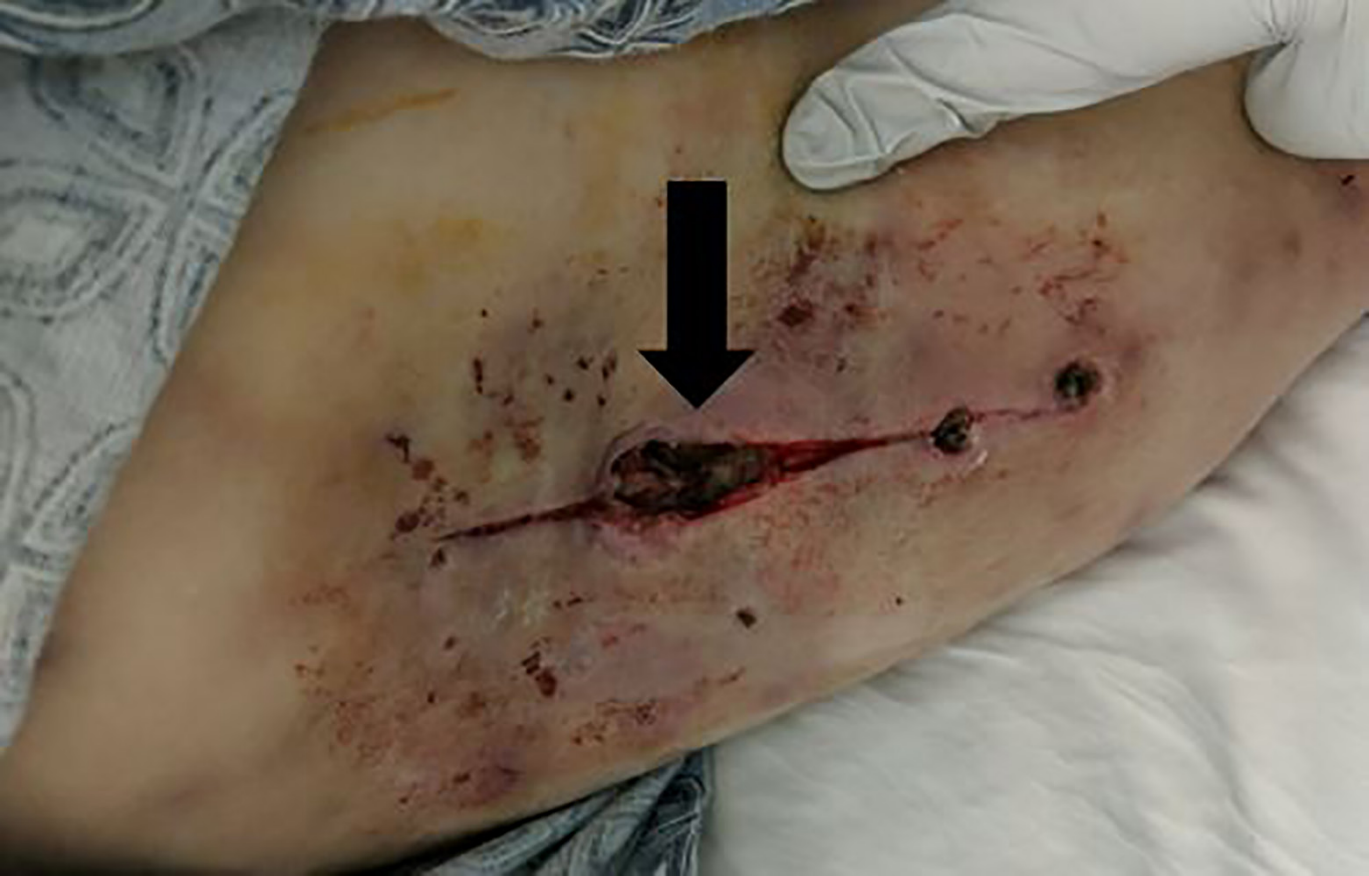
Deep right upper extremity wound from “skin popping.” Photo taken after debridement for wound botulism. Mild surrounding erythema noted.

The day that wound cultures were obtained the patient was started on empiric botulism anti-toxin and underwent surgical debridement and wound excision for source control (Images 2, 3). Simultaneously she was started on broad spectrum antibiotics. She exhibited mild clinical improvement but did require tracheostomy due to prolonged intubation and respiratory weakness. The patient remained in the hospital for approximately two weeks. Prior to discharge, she was determined to have such profound residual generalized motor weakness that she required transfer for rehabilitation and subsequent long-term care.

## DISCUSSION

Wound botulism can have a wide variety of presentations and is frequently misdiagnosed on the first healthcare encounter.^6^ If left undiagnosed, it can cause profound respiratory weakness leading to respiratory failure and the need for prolonged mechanical ventilation.^7^ While most cases present in clusters, this appeared to be an isolated case.^4^ It was particularly challenging as the patient’s primary symptom was isolated dysphagia, which was initially described as non-progressive and without other cranial nerve dysfunction. This could have been due to low levels of toxin production from a low-grade infection, as there were only minimal signs of cellulitis surrounding the wounds. She required multiple ED visits, hospital admission, and a prolonged ICU stay before a diagnosis could be established.

Patients suffering from substance abuse, drug intoxication or psychiatric illness are often seen in the ED. Emergency physicians evaluating these patients may be inclined to attribute subjective or subtle symptoms to the patient’s personality, drug intoxication, or underlying psychiatric illness. As a result, these patients have a propensity for leaving against medical advice and are at high risk of not being evaluated comprehensively. This can lead to multiple ED visits as it becomes easier for emergency physicians to overvalue previous non-diagnostic workups, thus continuing momentum toward a lack of an emergent diagnosis. Moreover, wound botulism is also because of its rarity.^8^ The combination of these biases can result in missed diagnoses of critical illnesses. Emergency physicians should be aware of these biases and keep them in consideration especially during high-risk patient encounters.

This case highlights some of the core tenets of wound botulism treatment, including botulinum anti-toxin and surgical source control. Whereas food botulism often responds to anti-toxin alone without surgical intervention, treatment of wound botulism with anti-toxin alone may not be successful.^7^ This is noteworthy, as case series demonstrate that time to anti-toxin and time to surgical debridement shortens hospital stays significantly.^3^ To obtain botulism anti-toxin or submit samples for testing, providers should contact their local state health departments, as well as the CDC at 770-488-7100.^9^

## CONCLUSION

Botulism, specifically wound botulism, presents a diagnostic challenge in the ED, especially when compounded with underlying social factors and possible cognitive biases. Providers should be aware that wound botulism can present with isolated cranial nerve dysfunction, may not initially be described as progressive, and that “typical” presentations are the exception, rather than the rule.
